# Women’s autonomy and men's involvement in child care and feeding as predictors of infant and young child anthropometric indices in coffee farming households of Jimma Zone, South West of Ethiopia

**DOI:** 10.1371/journal.pone.0172885

**Published:** 2017-03-06

**Authors:** Kalkidan Hassen Abate, Tefera Belachew

**Affiliations:** Department of Population and family health College of health Sciences, Jimma University, Jimma, Ethiopia; Western Sydney University, AUSTRALIA

## Abstract

**Background:**

Most of child mortality and under nutrition in developing world were attributed to suboptimal childcare and feeding, which needs detailed investigation beyond the proximal factors. This study was conducted with the aim of assessing associations of women’s autonomy and men’s involvement with child anthropometric indices in cash crop livelihood areas of South West Ethiopia.

**Methods:**

Multi-stage stratified sampling was used to select 749 farming households living in three coffee producing sub-districts of Jimma zone, Ethiopia. Domains of women’s Autonomy were measured by a tool adapted from demographic health survey. A model for determination of paternal involvement in childcare was employed. Caring practices were assessed through the WHO Infant and young child feeding practice core indicators. Length and weight measurements were taken in duplicate using standard techniques. Data were analyzed using SPSS for windows version 21. A multivariable linear regression was used to predict weight for height Z-scores and length for age Z-scores after adjusting for various factors.

**Results:**

The mean (sd) scores of weight for age (WAZ), height for age (HAZ), weight for height (WHZ) and BMI for age (BAZ) was -0.52(1.26), -0.73(1.43), -0.13(1.34) and -0.1(1.39) respectively. The results of multi variable linear regression analyses showed that WHZ scores of children of mothers who had autonomy of conducting big purchase were higher by 0.42 compared to children's whose mothers had not. In addition, a child whose father was involved in childcare and feeding had higher HAZ score by 0.1. Regarding age, as for every month increase in age of child, a 0.04 point decrease in HAZ score and a 0.01 point decrease in WHZ were noted. Similarly, a child living in food insecure households had lower HAZ score by 0.29 compared to child of food secured households. As family size increased by a person a WHZ score of a child is decreased by 0.08. WHZ and HAZ scores of male child was found lower by 0.25 and 0.38 respectively compared to a female child of same age.

**Conclusion:**

Women’s autonomy and men’s involvement appeared in tandem with better child anthropometric outcomes. Nutrition interventions in such setting should integrate enhancing women’s autonomy over resource and men’s involvement in childcare and feeding, in addition to food security measures.

## Background

Profound and widespread reductions in child mortality across the globe had been achieved through remarkable commitment of countries through the Millennium Development Goal 4 (MDG4)[1]. Yet, a number of lower income countries, particularly in sub-Saharan Africa, still experience high rates of child mortality [2]. According to The United Nations Children's Fund’s (UNICEF) estimate, in 2015 alone, 5.9 million under-5 children died, mostly as a result of problems or diseases that can be affordably prevented or treated [3]. At the epicenter of these deaths, nutritional problems certainly exist, especially in poor countries. Approximately, one in every thirteen children of the globe are wasted, while nearly a quarter are stunted, of whom more than 80% lived in Asia and Africa [4]. In Ethiopia, despite unprecedented achievement in reducing child mortality faster than what was anticipated through MDG period, the national prevalence of stunting (38%) and wasting (9%) still persisted to be high[5].

Child health, growth and development are results of multi layered factors that have direct or indirect causal links. The complex relationships among these factors have been best portrayed in the depiction of the UNICEF global conceptual framework of malnutrition [6]. In this framework, factors were analyzed in terms of immediate, underlying and basic causes of child malnutrition. The *immediate factors* presented were sub-optimal dietary intake and diseases reflecting the *underlying* social and economic conditions of the household. The underlying factors are depicted as a consequence of distal or *basic* determinants such as political, economic, and ideological structures within the community or the country. This frame work has most widely been used across studies and programs to uncover the intricate and multifaceted determinants of malnutrition for quarters of a decade.

Child susceptibility to failure of growth, morbidity and mortality from birth to two years is very high [7].Studies identified the fact that the first 1000 days of human life is a “critical window” for promotion of optimal growth, health and development [7–11]. The Optimal feeding recommendations at this time include adequate intake of macro and micronutrients during pregnancy, breastfeeding only for the first 6 months after birth and breastfeeding in combination with complementary foods from 6–24 months of age [8–11]. In Ethiopia, child malnutrition is mostly a reflection of poor child caring practices. Seventy percent of children under the age of five years are sub optimally breastfed, only 54 percent are exclusively breastfed during the first 6 months, while only 43 percent of children 6–9 months are optimally fed [12].Caring practices can be hindered by livelihood factors which possibly modify maternal access to resources for childcare, such as in cash cropping, where, income should be translated in to food prior to implementation of optimal child feeding [13–14]. Many studies conducted in Africa justified maternal concern over resources, as men tended to control income from cash crops and pay for lump-sum and prestige items than food [15–16].

In earlier reviews of studies, most domains of socio economic, maternal and child characteristics such as parental education, maternal age, maternal nutritional status, marital status, occupation and income, method of feeding, optimal initiation of complementary feeding, meal frequency, dietary diversity, child morbidity, sex and age were identified and reported as predictors of child nutritional outcome [17–20]. However, women’s autonomy, which is a possible factor influencing childcare and nutritional outcomes was least studied. The theoretical rationale that links women’s autonomy and child malnutrition is dual such that it is either through the pathway of maternal own nutritional status which affect breast feeding or through reduction of access to household resources for childcare [21]. There are subtle evidences that suggest women who have lower autonomy within their household suffer from under nutrition themselves [22–24].Most domains of women’s autonomy (their decision making on household asset, their own and their children) were integral part of demographic health surveys (DHS) assessment tools of developing countries. Some notable reports of DHS data on effect of women’s autonomy on nutritional outcome of children were those done for Bangladesh and Tanzania [25–26].The study in Bangladesh showed that children whose mothers participated in household decision making were 15%, 16%, and 32% less likely to be stunted, underweight, and wasted, respectively than mothers who did not participate in decision making. Similarly, the result of a study in Tanzania revealed children who belonged to mothers with autonomy of decision making on their own healthcare had better nutritional outcome compared to children whose mothers did not have the autonomy.

Meanwhile, available reports of other studies like the “Asian Enigma” of Ramalingaswami *et al* described women’s status accountable for the differences in the prevalence of stunting between Africa and South Asia, where the latter has more stunted children despite its economic superiority over the former [27]. Similarly, Monal *et al*.(2009),showed two dimensions of maternal autonomy (financial and mobility) as an independent predictors of childcare and stunting of children in Andhra Pradesh, India[28].The only available systematic review to our knowledge reported by Carlson et al. (2015), strongly suggested that raising maternal autonomy is a key intervention for improving children’s nutritional status [29]. Despite all the facts discussed above, maternal autonomy does not always have a direct or positive correlation with childcare and or nutritional outcome. Studies in Kenya and Nepal showed that maternal autonomy variables have a limited or no influence on child nutrition measures [30–31]. Rajaram*et al* (2016), also showed a statistically insignificant association between women’s autonomy in any form (healthcare, or movement, or money) and child nutritional outcome [32]. Similarly, Rushdie (2004), specifically documented no statistical association between women’s access and decision making over cash resources and stunting of children [33]. Negative than mostly anticipated, Smith *et al*(2003) reported that an increase in decision-making power of women associated with decreased exclusive breastfeeding, decreased breastfeeding duration and increased bottle-feeding, reflecting the complex nature of the relationship, between autonomy and care [34].

Mostly, childcare and feeding has been regarded as female’s domain and majority of researchers have focused on the role of this liaison on health and nutritional outcome of children. The role of the father, though acknowledged, is the most neglected part in the continuum of child health care and research process of developing world so far. In low income countries, a child health care is ‘mother centric’, and less effective in participating the father [35]. Policy directions were not lacking, as paternal involvement was discoursed and honed in maternal child health care activities since the 1994 International Conference on Population and Development (ICPD) [36]. The mounting concerns of women’s morbidity and mortality in low-resource settings and concurrent infant and child health issues had been the driver of the initiatives to involve men in the Cairo meeting. Available studies done to assess effects of paternal care on child nutritional and health outcomes have employed two approaches, either through analyzing gains of fathers’ involvement or gaps due to his absence. Methodologically, results of studies designed on assessing effect of father’s involvement in childcare were more pragmatic in public health action compared to those studied on the effects of fathers’ absence on child outcomes [37].

Albeit few, there also exists affirmative findings on paternal involvement that have positive impact on child nutritional outcome. A Vietnamese study showed that children whose fathers did not bring them to the medical facilities for immunizations were about 1.7 times more likely to be malnourished which indicate the need for paternal involvement in child health care system in general and nutritional outcome in particular [38]. Similarly, a study done South Africa reported children whose fathers did not provide their family with financial support were found to be at higher risk of malnutrition [39]. On the psychosocial aspect of childcare, the fathers’ role are also important. Dearden*et al* (2013) reported lower HAZ scores among children who did not see their fathers on a daily or weekly basis during their infancy and childhood compared to children who saw their fathers regularly, after adjusting for maternal age, wealth and other contextual factors [40]. A sub-Saharan Africa study also corroborates the above findings. According to the study, higher odds of stunting among children of single mothers were reported compared to children whose mothers were in union [41].Furthermore, findings from Jamaican study also showed children from single-parent homes or cohabiting households are at higher risk of under nutrition irrespective of income [42].

In Ethiopia, thus far we could not find any study similar to our objective. Moreover on coffee farming population such studies are lacking. Thus, we set out to document the association between maternal autonomy, men’s involvement in childcare and feeding with nutritional status of children in coffee farming households of Jimma Zone, Southwest Ethiopia, in order to generate evidence on those context specific factors.

## Research design and methods

### Study setting and design

A community based cross-sectional study was conducted on Infant and young Childs of coffee producing households of Jimma Zone, Southwest Ethiopia. Jimma zone is one of the 18 zones of Oromia region which is believed to be the birthplace of Coffee [43]. Organic coffee of Jimma zone is the backbone of foreign exchange of the country, which accounts for 4.2 percent of the total world coffee production, sustaining 15 million Ethiopians in its economic chain [44]. According to 2007 national census, the total population and households of the zone were 2,495,795and 521,506 respectively. This zone covers a total area of 15,569 Km2, with reliable rain fall ranging from 1,200–2,800 mm per annum [45–46].

### Sample size and sampling procedure

The Sample size for the study was calculated using a prevalence of malnutrition in Mana Woreda of Jimma Zone (42%), a design effect of 2 and a margin of error of 0.05 [47]. A total sample size of 749 was estimated to have a power of 80, calculated using Epi info Version 7 open source sample size calculator. The inclusion criteria was being an infant or young child of permanently registered resident farming household of the Woredas, while exclusions were made on children with severe acute malnutrition warranting referral to nutrition rehabilitation program, severe illness with clinical complications warranting hospital referral and presence of obvious congenital or chronic abnormalities that impair feeding or physical growth measurements. Multi-stage stratified sampling was used to collect data from respondents across the zone. First, three of the nine top coffee producing Woredas of Jimma zone (Mana, Gomma and Limukossa) were randomly selected. Then, the Woredaswere stratified by urban and rural areas of residence and finally one third of villages (Gots) in rural areas and kebeles in urban setting were selected and used as primary sampling unit, followed by a random selection of households with young infant and young child using health extension workers’ registry as a frame. The sample size in each stage was allocated based on proportional to size allocation methods based on central statistics agency report of 2007 [45]. In the event where more than one eligible child was found in a house, the youngest was taken.

### Data collection and procedures

A structured questionnaire was used for face to face interview of mothers/caregivers. The two immediate causes of malnutrition, inadequate dietary intake and diseases were assessed by dietary methods and morbidity reports respectively. Exclusive breast feeding under the age of 6 months and dietary diversity with feeding frequency for 6–24 months of age children were used as a proxy measure of optimal feeding. The three underlying causes of malnutrition food access, hygiene and childcare were assessed using household food insecurity scale (HFIAS), diarrheal morbidity report (as a proxy indicator of hygiene)and the WHO Infant and young child feeding (IYCF) practice core indicators respectively. All values to indicate optimal practice were based on age specific guideline of WHO and their compliance is considered as optimal childcare and feeding [8–11]. The basic factor for optimal nutrition is assessed by collecting data on socio-demographic variables, households’ assets and utilities, maternal, paternal and child characteristics. The interview were made by trained nurses while anthropometric measurements were taken by three trained graduate nutritionist. Ethical clearance was obtained from the institutional review board of collage of Health sciences, Jimma University, Ethiopia. Respective government and health institutions and local administrators were requested for permission of entry using an official letter from the university. Detailed description of the study to Kebele and “Got” leaders and households were provided while separate informed verbal and written consent for each study participant were obtained.

### Measurements and analysis

Women’s autonomy was measured by four theoretical proxy domains adapted from DHS tool; ‘*mobility*’, ‘*decision regarding child*’, ‘*decision regarding family planning*’ and ‘*finance*’. We inquired the mother eight items with binary ‘yes’ or ‘no’ answers, where, ‘0’ represented a no autonomy or involvement and ‘1’ represented a higher level. The first three questions were related to ‘*mobility*’, asking the mother if she required approval from her husband or family member to go to ‘*outside home*’, or ‘*market place*’, or ‘*health institution*’. The next three questions were related to ‘*mother involvement in decision making regarding her child’;* specifically, ‘*when child got sick*’, or ‘*child schooling*’ or ‘to *whom to marry*’. The third group of questions related to ‘*financial autonomy*’ inquiring mothers autonomy on ‘*purchase of food*’ or ‘*big item such as oxen*, *land and house*’. We also asked a single item on autonomy of ‘*utilization of family planning service*’. Similarly, Paternal involvement in childcare is assessed by five theoretical proxy domains drawn and adapted from Lamb et al., (1987); ‘*presence’*, ‘*engagement in care’*, *‘finance’*, ‘*child health care seeking’* and ‘*informational role’*[48].Among the above paternal involvement variables ‘*paternal engagement in care*’ was assessed by two questions. The first item inquired whether the father had ‘*engaged in feeding’* of his child. The second question probed the father ‘*engagement in child hygiene and psychosocial support’ such as diapering*, *bathing*, *handling and playing*. Affirmative responses for both questions were set as criterion for a father with a child of 6–24 months of age for ‘*optimal paternal involvement in childcare* ‘.For those fathers with a child 0–6 month’s age, only the second question was taken as a criterion. ‘*Paternal presence*’ was determined by calculating the ratio of months at which the ‘father lived with the child in the same roof’ to ‘the child age’. ‘*Paternal involvement in child health care seeking*’ was assessed by asking the father ‘*if he ever brought his child to health institutions since his birth’*. Meeting “*informational role of the father “*was assessed by asking the mother *‘if she had ever received information about optimal childcare from the father of her child or not’*.

Household Food Insecurity Access Scale (HFIAS) version 3 was used to measure household food security status. HFIAS has been developed by FAO and Food and Nutrition Technical Assistance (FANTA) and validated for use in Ethiopia [49]. Though adaptation for local context is highly recommended in different studies, we used the tool as it is (without change) for the benefit of its ascertained validity and reliability in Ethiopia [50–51]. The instrument has nine items categorized in three domains, anxiety and uncertainty, Insufficient Quality and insufficient food intake and its physical consequences. Definitions of the HFIAS instrument were used to label households as food secure or insecure [49]. Dietary diversity of children was measured using FANTA tool as recommended by the WHO Infant and young child feeding (IYCF) recommendations guideline [8–10]. Optimal achievement of minimum dietary diversity was defined as proportion of children with 6–23 months of age who received foods from four or more food groups of the seven food groups. The seven foods groups used for tabulation of this indicator were adapted for local food items. For example we added “Teff” a local cereal in the grains list of the probing instrument. Consumption of any amount from each food group was sufficient to ‘count’, i.e., there was no minimum quantity, except if an item was only used as a condiment (S1 File). In the same manner, we adopted the WHO IYCF feeding recommendation definitions to assess children’s achievements for Minimum meal frequency [8–10]. Accordingly, the Minimum frequency was defined as proportion of breastfed and non-breastfed children aged 6–23 months who received solid, semisolid, or soft foods twice for breastfed infants 6–8 months, three times for breastfed children 9–23 months, and four times for non-breastfed children 6–23 months.

Length and weight measurements were taken in duplicate using calibrated equipment and standardized techniques. Length [height] was measured in the recumbent position to the nearest 0.1 cm using a measuring board with an upright base and movable headpiece made by Seca, Germany. Weight was measured using weighing scales (Seca, Germany) (+10 g precision) with light clothing. Data were entered into EpiData to control skip patterns and allow double entry and exported to SPSS version 21 for analysis. Anthropometric data were analyzed using WHO Anthro version 3.2.2. In the analysis, plausibility of anthropometric Z scores were checked using the WHO protocol recommendations (2006), which provide standard deviation cut points for anthropometric Z-scores as a data quality assessment tool [52].Accordingly, implausible z scores data were excluded if a child’s HAZ was below –6 or above +6, WAZ below –6 or above +5, WHZ below –5 or above +5, or BMIZ below –5 or above +5.

Wealth index was generated using Principal Components Analysis (PCA).The scores for 25 types of assets and utilities were translated into latent factors and the first factor that explained most of the variation was used to group study households into wealth tertile. Each question on domains of autonomy and men’s involvement were summed up for their category to generate count base index. Under nutrition were defined based on their indices including: weight-for-age Z-score (WAZ), height-for-age Z-score (HAZ), weight-for-height Z-score (WHZ) and BMI for age Z-score (BAZ). The World Health Organization Child Growth Standards were used to classify nutritional status [53]. Accordingly, children whose weight-for-age z scores was less than -2 SDs below the median for their age and gender were defined as being underweight. Children with height-for-age z scores less than -2 SDs below the median were defined as being stunted and those with weight-for-height Z scores less than -2 SDs below the median was considered as wasted. Severe anthropometric failure is also defined as less than -3 SDs below the World Health Organization determined median scores for each indexes. A multiple linear regression was conducted to isolate independent predictors of nutritional outcomes of child using SPSS version 21 windows software.

## Results

Most of the households were residents of the rural area (87.7%). The majority was Muslims (82.4%) and Oromo ethnic group (76.5%). Most of the interviewed heads of the households were married (91.5%) and almost equally headed by male gender (90%). The mean family size in the studied households was 5.1 with standard deviation of (SD) ±1.8. The mean (SD) age dependency ratio was 0.5± 0.2. Quarter (25.2%) of the households was in the lowest tertile of the wealth index of the studied population, while comparable proportions of households were found in upper and middle tertile. Majority (87.7%) of the households had less than one hectare farm land. Prevalence of food insecurity in the setting was 68.8% (Table 1).

**Table 1:** Socio-demographic characteristics of coffee producing Households, Jimma Zone, Ethiopia, 2016.

Variables N = 749		N(%) or Mean± Sd
Setting	Rural	657
	Semi Urban	92
Religion	Muslim	617
	Orthodox Christians	113
	Protestant	19
Ethnicity	Oromo	573
	Amhara	60
	Silte	55
	Dawero	49
	Others	21
Marital Status	Married	685
	Divorced	53
	Widowed	11
Sex Of The Household Head	Male	674
	Female	75
Food insecurity	Insecure	515
	secure	232
Wealth Index	Higher	293
	Lower	185
	Medium	271
land Size	<1 Hectares	657
	>1 Hectares	92
Family Size		5.1±1.8
Mean of Age Dependency ratio		0.5±0.2

The median, mean and standard deviation (SD) of mother age were 25, 26.7 and 5.4, respectively. The age range of the mothers was15-44 years, few (2.8%) were underage groups (i.e. below 18 years). The median, and mean (SD) of age at which the mothers married were 18 and 18.4 (3.1) respectively. The median, mean (SD) age difference of couples was 7 and 7.7 years respectively, and ranging from -10 to 40 years. Regarding educational status, more than two third (68.2%) of the mothers have attended formal education.

Most mothers (60.5%) required ‘permission or approval to go outside their home’ (mobility autonomy). Almost equal proportion of mothers reported permission requirements to visit the local health institution (61%) and local market (60.2%). Regarding decision making autonomy, the majority of them gave affirmative responses for ‘when child got sick’ (73.7). Conversely, the proportion were reduced to two third and half when it comes to ‘child schooling’ and ‘to whom to marry a child’ respectively. Around 80% of the mothers responded ‘yes’ when asked ‘if they need approval or permission to work out side home’. More than half (56%) responded that they have autonomy of decision making related to food purchase. Conversely, only 44% of mothers were reported to have autonomy in conducting big purchases. A very low level of autonomy was reported regarding utilization of family planning service (37.8%).

Regarding nutritional status of the mother, the median and mean (SD) of body mass index of the mother were 20.3, 20.8(3.1), respectively. Few (1.7%) of the mothers were pregnant with median, mean (SD) of middle upper arm circumference (MUAC) 23, 22.3(3.5) respectively. The prevalence of underweight among mothers (BMI<18.5) or MUAC<23 were 24.2%, while the proportion of overweight and or obesity were 10% (Table 2).

**Table 2:** Frequency distribution of domains of maternal autonomy, male involvement and child characteristics of among coffee farming households of Jimma Zone.

Maternal Characteris**ticsN = 749**	Variables*		N or Mean ±SD
	Maternal Age	In years	26.7**± 5.4**
	Maternal education	Formal education	511
		No formal education	238
	Maternal BMI	Kg/M^2^	20.8**±**3.1
	Freedom of Movement; seeking permission to go to;	Outside home (yes)	453
		Market place (yes)	451
		Health institution (yes)	457
	Maternal involvement indecision regarding child;	Sickness	552
		Schooling	496
		To whom to Marry	406
	Maternal Autonomy in conducting;	Food purchase (the mother involved)	419
		Big Item Purchase (mother is involved)	328
	Autonomy regarding Family planning service utilization	yes	283
	Maternal Age at first marriage	In years	18**±3.0**
	Father-mother age difference	In years	7.7**±6.0**
Paternal CharacteristicsN = 749	Age of father	years	34.4 ±8.4
	Educational status	Formal education	583
		No Formal education	166
	Paternal Involvement in Child care	Feeding (n = 548)	427
		Other care (hygiene and psychosocial))	599
	Paternal Involvement: Financial	Yes	686
	Paternal Involvement: Child Health care seeking	Yes	264
	Paternal Involvement: Presence	Mean of the ratio of father and child lived in the same house	0.92**±0.25**
	Paternal Involvement: Informational	Yes	377(50.3)
**Child characteristics**	Age	Months	12**±**7.6
	sex	Male	410
	Optimal child feeding indicators	Exclusive breast feeding	232
		Optimal Dietary diversity (n = 548)	315
		Optimal MMF (n = 548)	277
		Minimum acceptable diet (n = 548)	93
	Morbidity last 4 weeks	Diarrheal	33
		Acute respiratory illness	173
		Eye infection	14
		Ear discharge	10
		Skin rash	16

*N = 749 unless specified.

The median and mean (SD) of fathers age were 32, 34.5(8.5) years, respectively. Paternal age range was 19–80 and more than 11% of the fathers were in their elderly. Most of the fathers (77.8%) have attended formal education. As to their involvement in childcare, most of them gave affirmative responses for childcare (80%), finance (91%) and feeding (78%). On contrary, their engagements in health institution for their child health purpose and informational role were lower, 62% and 50.3%, respectively (Table 2).

The median and mean (SD) age of child were 13, 12(7.6) months respectively. Most of them (73%) were above 6 months of age. More than half 54.7% were male. Proportion of exclusive breast feeding during the first six months of the child age was nearly 31%. However, children ever breastfed were (98.9%). The proportion of children who had optimal dietary diversity measured by having four or more food groups out of seven was 42%. Attainment of minimum meal frequency as defined by proportion of children 6–23 months of age, who received solid, semi-solid, or soft foods with the minimum number of times or more was 37%. The proportion of children meeting the definitions of optimal infant and young child feeding indicators of WHO was 27.5% (Table 2).

The mean (sd) scores of weight for age (WAZ), height for age (HAZ), weight for height(WHZ) and BMI for age (BAZ) was -0.52(1.26), -0.73(1.43), -0.13(1.34) and -0.1(1.39) respectively. The prevalence of wasting and stunting were 8.8% and 19.7% respectively. The proportion of moderate acute malnutrition (MAM) was 5.7%, while severe acute malnutrition (SAM) was 3.1%. Moderate form of chronic malnutrition was 12.6% while severe stunting was 7.1%. The prevalence of diarrheal and acute respiratory illness (ARI) were 4.4% and 23% respectively (Table 3, S1 Table).

**Table 3:** Anthropometric Z-scores of Infants/young Childs of Jimma Zone, South west Ethiopia, 2016.

Z scores	Age groups	N	% < -3SD	(95% CI)	%< -2SD	(95% CI)	Mean	SD	Median	Range(Min, Max)
**Weight-for-length**	(0–5)	193	4.1	(1.1%, 7.2%)	10.4	(5.8%, 14.9%)	-0.08	1.48	0.04	(-4.87,3.28)
	(6–11)	175	3.4	(0.4%, 6.4%)	9.1	(4.6%, 13.7%)	-0.25	1.37	-.23	(-4.6, 3.3)
	(12–23)	363	2.5	(0.7%, 4.2%)	7.7	(4.8%, 10.6%)	-0.11	1.25	.01	(-4.3,3.7)
	Total:	731	3.1	(1.8%, 4.5%)	8.8	(6.6%, 10.9%)	-0.13	1.34	0.002	(-4.8–3.7)
**Height-for-length**	(0–5)	193	5.2	(1.8%, 8.6%)	16.1	(10.6%, 21.5%)	-0.36	1.45	-0.01	(-4.6, 3.41)
	(6–11)	175	4.6	(1.2%, 8%)	11.4	(6.4%, 16.4%)	-0.35	1.37	-0.23	(-4.9,2.2)
	(12–23)	363	9.4	(6.2%, 12.5%)	25.6	(21%, 30.2%)	-1.12	1.34	-1.15	(-4.8,1.9)
	Total:	731	7.1	(5.2%, 9%)	19.7	(16.7%, 22.7%)	-0.73	1.43	-.55	(-4.9,3.4)

On multivariable linear regression model, the WHZ scores of children of mothers with who had the autonomy of conducting big purchase were higher by 0.42 compared to children's whose mothers had not. In addition, a child whose father involved in childcare was found to have a higher HAZ score by 0.1. Regarding age, as for every month increase in age of child, a 0.04 point decrease in HAZ score and a 0.01 point decrease in WHZ were noted. Similarly, a child living in food insecure households had lower HAZ score by 0.29 compared to child of food secured households. WHZ and HAZ scores of male child was found lower by 0.25 and 0.38, respectively compared to a female child of same age. Optimally fed children were found having higher Z score by 0.28. As family size increased by a person a WHZ score of a child is decreased by 0.08. (Table 4, S2 Table).

**Table 4:** Determinants of WHZ and HAZ scores of Infants/young Childs of Jimma Zone, South West Ethiopia, 2016.

	**Weight for height Z scores**			**Height for age Z scores**		
**Variables**	Standardized Coefficients	Sig.	95%CI	Standardized Coefficients	Sig	95%CI
Setting	.06	.69	(-.25,.37)	-.12	.46	(-.44,.20)
Family size	-.08	.03*	(-.15,-.01)	.02	.58	(-.05,.09)
Sex of household head	.09	.70	(-.35,.53)	-.15	.53	(-.60,.31)
Maternal age	.01	.25	(-.01,.04)	.01	.41	(-.01,.03)
Sex of the child	-.25	.01*	(-.45,-.05)	-.38	.00*	(-.58,-.17)
Household food insecurity	-.07	.54	(-.29,.15)	-.29	.01	(-.51,-.06)
Child Age	-.01	.44	(-.02,.01)	-.04	.00*	(-.05,-.03)
Maternal Autonomy: Mobility	.00	.90	(-.07,.08)	.03	.45	(-.05,.11)
Maternal Autonomy: Decision regarding Child	.03	.57	(-(.06,.11)	-.03	.45	(-.12,.06)
Maternal Autonomy: Food purchase	.15	.27	(-.12,.43)	.11	.44	(-.17,.39)
Maternal Autonomy: Big Item Purchase	.42	.00*	(.16,.69)	.03	.81	(-.24,.31)
Maternal Autonomy: Family planning	-.03	.79	(-.27,.21)	-.01	.93	(-.26,.24)
Paternal Involvement in Care	-.13	.47	(-.49,.22)	.42	.02*	(.05,.79)
Diarrheal Morbidity	.19	.43	(-.28,.66)	-.30	.23	(-.78,.19)
Maternal Age at first marriage	.00	.78	(-.04,.03)	.01	.52	(-.02,.05)
ARI	-.22	.06	(-.46,.01)	-.12	.35	(-.36,.13)
Optimal Child feeding	.03	.82	(-.20,.25)	.28	.01*	(.06,.51)
Marital status	.22	.22	(-.14,.58)	-.22	.24	(-.60,.15)
Land size	.14	.16	(-.06,.34)	-.20	.05	(-.41,.00)
Spousal education	.17	.13	(-.05,.38)	-.16	.16	(-.39,.06)
Wealth index	.05	.41	(-.07,.18)	-.05	.47	(-.18,.08)
Dependency ratio	-.18	.48	(-.69,.33)	-.05	.86	(-.57,.48)
Household head education	-.05	.70	(-.29,.19)	-.10	.43	(-.35,.15)
Maternal BMI	-.01	.58	(-.04,.02)	.00	.87	(-.04,.03)

*Significant.

## Discussion

The current study assessed the association of women’s autonomy and men’s involvement with child nutrition, adjusted for dietary, health and socioeconomic variables. The prevalence of wasting and stunting were very high, 8.8% and 19.7% 10% respectively. The finding on wasting prevalence was a bit lower than the present (2016) Ethiopian DHS report (9%) as well as the earlier (2011) (9.3%) [5, 12].This difference could be due to the tendency of wasting for changes over a short duration and differences in the season of measurement. Unlike wasting, a significantly lower level of stunting (19.7%) was documented compared to the above DHS reports [5, 12]. The lower level of stunting could be explained by the differences in agro-ecologic advantage of the Jimma Zone [44–46]. However, the findings of this study were higher than major coffee producing developing regions of Latin America and the Caribbean where regional prevalence of underweight, wasting, and stunting were 4%, 2%, and 15%, respectively [54]. In line with our anticipation, optimal feeding was found as one of the determinants of HAZ score. Optimally fed children were found having higher Z score by 0.28. There have been similar finding reported across studies in Ethiopia [2, 12, and 55]. However, optimal feeding showed no significant association with WHZ scores which could be attributed to the nature of WHZ scores, acquiescent for acute changes in diet or health.

Attainment of women’s autonomy indicators in the study setting was not ideal. The autonomy domain attained lowest was “decision making regarding family planning utilization” (37%), while ‘maternal decision making autonomy on sick childcare’ was attained most of mothers (73%). Most of the domains of maternal autonomy studied showed non-significant association with child Anthropometric Z scores. The only exception is “maternal autonomy regarding conducting big purchase”, which became one of the determinants of WHZ scores of children. Children whose mothers had autonomy of conducting big purchase were found having higher WHZ score by 0.42 compared to children whose mothers had not. This finding is in line with other studies which reported less odds of low birth weight and higher WAZ score among child whose mothers had autonomy of conducting big purchase [28, 31].It also corroborates findings of Smith et al., (2003)who showed higher decision making power including finance positively associated with child WAZ scores [34]. Higher financial decision making power give mothers the ability of managing acute dietary or health assaults early before nutritional course of the child changed to the worst. Lack of association of other domains of women’s autonomy and child nutritional outcome could be due to the typical approach of this study which adjust women’s autonomy by men’s involvement, not the trend employed in other similar studies. Furthermore, it could be due to additional cross cultural factors which could be beyond the scope of this study and limitations associated with our tool in measuring autonomy which involve socially sensitive inquiries.

Realization of paternal involvement in the study setting were higher in terms of *presence*, *finance* and *childcare and feeding* but lower in *child health care seeking* and *informational role*. Surprisingly, paternal engagement in childcare and feeding was found as one of the determinant factors for HAZ scores. This could be due to additional care the child gained through the father. Even though establishing causal association is very difficult considering the complexity of the subject, one can hypothesize the positive outcome of this liaison as indicative of the existing family integrity. When the father is involved, positive child rearing environment is established through reinforcing positive motherhood behavior. Review of this positive feedback relationship reports were best summarized by Allen and Daly (2007) [56].

Male children in the study area showed lower anthropometric indices in contrast to their counterparts. A similar phenomenon was observed in the demographic and health surveys of the country (DHS) [5, 12]. In sub-Saharan Africa, male children under the age of five were more likely to become stunted than females. This might suggest that boys are more vulnerable to nutritional inequalities than their female counterparts of the same age groups [57]. There is high level of food insecurity in the study setting, showing inverse statistical association with HAZ scores of children. Belachew et al., (2013) also showed similar relationship in the same study area but for adolescents [58]. Family size in the study was found to have inverse relationship with WHZ scores of children. The inverse association has been often reported in the DHS reports of the country [5, 12]. Large family size may hamper the mothers’ potential for optimal feeding practices. A recent studies in Ethiopia and Nigeria indicated low appropriate complementary feeding practice of children in families with larger family size [59–60]. Furthermore, in their economic review, Filmer et al, (2009) also suggested larger family size may put children at higher risk for acute malnutrition, which could be due to the imbalance between family size and resources [61]

In the current study, the most likely factor “maternal education” was not statistically significant. Under most circumstance, education empowers women through employment and earnings. When a women has access to resources, mainly earnings, her potential to assume positive child caring behavior is improved, yielding better nutritional environment for children. However, in the current setting their inability to make higher financial decisions (limited say in “big purchase”) may weaken the association of maternal education on child outcome. Contrary to the current finding, the existing abundant empirical literature reported the otherwise [5, 12, 17–19, 62]. Paternal education, maternal age, maternal BMI, father-mother age difference, marital status, Education empowers the mother through employment and earnings enhancing her bargaining power in the house. Dependency ratio, land size and child morbidity also failed to show statistical significance. Contrary to the classic relationship, wealth index showed statistically insignificant association with the child nutritional outcome. In fact such phenomenon justify the need for understanding context specific determinants in health and nutrition studies and interventions across settings [63]. In support of the current finding on wealth index, studies also showed nutritional status of households measured by anthropometric indices and health outcome did not necessarily improve with wealth or income [16, 64–65].

## Conclusion

Women’s autonomy and men’s involvement are in harmony with good anthropometric outcomes. The child anthropometric indices were found affected by the studied women’s as well as men’s intrinsic factors. Both maternal autonomy (for WHZ) and paternal engagement in care (for HAZ) were found determinants of child nutrition in the setting. Furthermore, optimal feeding, sex of the child and food insecurity were also remained significant predictors. Thus, nutrition interventions in such setting should integrate enhancing women’s autonomy over resource and Men’s involvement in childcare and feeding, besides food security measures.

### Limitation of the study

Many factors may likely underpin these findings, and, they should be thoroughly examined in future longitudinal and qualitative studies. Based on our current analysis, we cannot reliably parse out and attribute our findings without mentioning the validity of our instruments. Though the DHS and Lamb et al., approaches are widely used, the inherent difference across territories may hamper their sensitivity and specificity in measuring such complex variables. Available systematic reviews on such topics addressed the need for optimal clarity, reliability and validity of tools used for intrinsic variables such as autonomy and empowerment in different population groups and settings [66–67]. Thus we acknowledge our efforts to measure and quantify women’s autonomy and or men’s involvement in care may have been affected by methodological constraints. The problem of measurement and interpretation arises because these variables cross cultural and multidimensional interpretation.

## Supporting information

S1 File Questionnaire on Household Characteristics, Women’s Autonomy and Men's Involvement in Child Care and Feeding of Infant and Young Child of Jimma Zone, 2016. (DOCX)

S1 Table Nutritional survey analysis of Infants/young Childs of Jimma Zone, South west Ethiopia, 2016. (XLSX)

S2 Table Multivariable linear regression statistics on factors affecting WHZ and HAZ scores of Infants/young Childs of Jimma Zone, South West Ethiopia, 2016. (XLSX)
