# Supplementary material for: Women’s autonomy and men's involvement in child care and feeding as predictors of infant and young child anthropometric indices in coffee farming households of Jimma Zone, South West of Ethiopia
Source: PLoS One. 2017 Mar 6;12(3):e0172885. doi: 10.1371/journal.pone.0172885 (PMC5338789; doi:10.1371/journal.pone.0172885)
Supplement: S1 File — (DOCX) [file pone.0172885.s001.docx]

# Questionnaire

***Instructions: The key respondent should be the mother of the selected index child, unless specified for the father.***

#

# Section A: Household Information

| Name | Variable | Response |
| --- | --- | --- |
| A1 | Date of interview |  |
| A2 | Setting | 1=rural 0= urban |
| A5 | Kebele | _________________________ |
| A6 | Village Gote |  |
| A7 | Household ID |  |
| A8 | Family size of the respondent | Persons |
| A9 | How many members of the household are below age 15 and above 65? |  |
| A10 | How many members of the your household are between the age b/n 15-65 |  |
| A11 | Marital status of the respondent | 1. Single. 2. Married  3. Widowed 4. Divorced |
| A12 | what is your marital form | 1. Mono 2. Polygamy |
| A13 | Sex of the household head | 1. Male 2. Female |
| A14 | Household head’s Educational status | 1=no formal education  2=primary education  3=secondary education  4=college/University |
| A15 | Spouse’s education | 1=no formal education  2=primary education  3=secondary education  4=college/University |
| A16 | Age of Father | ________Years |
| A17 | Age of mother | ________Years |
| A18 | Age at first marriage (mother) | ___________________Years |
| A19 | Age at first birth (mother) | ___________________Years |
| A20 | Age difference between spouse (years) | ___________________Years |
| A21 | Ethnicity |  |
| A22 | Religion |  |

**Section** B**. Household Assets**

| ***Now I will ask you about some fixed assets that your household have*.** | | | |
| --- | --- | --- | --- |
| Does the household have any of the following properties? **(Circle)** | | **Yes** | **No** |
| B1 | Functioning radio/Tape recorder/CD player | 1 | 0 |
| B2 | Functioning Television | 1 | 0 |
| B3 | Gas Stove | 1 | 0 |
| B4 | Kerosene stove | 1 | 0 |
| B5 | Electric stove | 1 | 0 |
| B6 | Bicycle | 1 | 0 |
| B7 | Motor Cycle | 1 | 0 |
| B8 | Cart/Gari | 1 | 0 |
| B9 | Watch (Hand/Wall) | 1 | 0 |
| B10 | Mobile phone | 1 | 0 |
| B11 | Plough | 1 | 0 |
| B13 | Sofa | 1 | 0 |
| B14 | Spring mattress | 1 | 0 |
| B15 | Sponge/Foam mattress | 1 | 0 |
| B16 | Cotton mattress | 1 | 0 |
| B17 | Grass Mattress | 1 | 0 |
| B18 | Chair/Stool | 1 | 0 |
| B19 | Generator | 1 | 0 |
| B20 | Milling | 1 | 0 |
| B21 | Water pump | 1 | 0 |
|  | **Does the household have any of the following animals?** | **1.Yes 0. No** | **How many?** |
| B22 | Oxen |  |  |
| B23 | Cows |  |  |
| B24 | Horse/mules/donkey |  |  |
| B25 | Goats/Sheep |  |  |

**Section C- Child Care and feeding**

| **Breastfeeding Advice** | | **Responses** |
| --- | --- | --- |
| C1. What have you heard or what advice have you received about breastfeeding? (Probe) | | 1. Exclusive up to six month 2. Feeding at least 8 or more times 3. Early initiation 4. Colostrum advantage 5. Other____________________ |
| C2. Who has talked to you about breastfeeding? | | 1. Health workers 2. Media 3. Neighbor hood 4. Others________ |
| C3. Has your husband ever given you advice about breast feeding | | 1. Yes 2. no |
| **Putting to the breast** | |  |
| C4. Was your child put to breast right after the birth? | | 1. Yes 2. No |
| C5. How soon after giving birth did the baby first receive breast-milk? | | 1. Immediately 2. 30 minutes later 3. One hour later 4. Others______________ |
| C6. Did your baby receive anything else before receiving breast-milk? | | 1. Yes 2. No |
| **Colostrum** | |  |
| C7. Did the baby receive the first milk (colostrum)? | | 1. Yes 2. No |
| C8. Has your husband ever given you advice about Colostrum? | | 1. Yes 2. No |
| **Breast-feeding pattern** | |  |
| **How many times do you breast feed your child in one day (24 hours)** | | **_____________________________** |
| C9. How long do you plan to continue to breastfeed? | | ------------ |
| C10. How long do you think a baby should be breastfed? | | -------------------------- |
| C11. For how long have you breastfeed the child thus far? | | 1. ---------------months  2. I stopped breast feeding  ________________ |
| C12. If Breast feeding stopped when did you stop breastfeeding? ( age of child month) | | __________________ |
| **For mothers already began with Introduction of foods** | | |
| C13. At what age did you start giving other liquids or food to your baby? | ____________ | |
| C14. What was/ were the food/s you started with? | 1. Porridge of cereal 2. Bread/Injera   3. Cow milk 4. Potato  5. Formula 6. Others__________ | |
| C15. Why did you choose those particular foods or liquids? | 1. Availability 2. Cost 3. Nutrient adequacy 4. others | |
| C16. How many times do you give your baby the above liquids/foods per day?  Probe. | 1. Once per day 2. Twice per day 3. Every other day 4. Others________ | |
| C17. How many times do you give your baby the above liquids/foods in the previous day?  Probe. |  | |
| C18. Which one of the following food item do you give your baby during the previous day?  * Use the probing tool attached to clarify the food groups  * Ask each item, put an X mark if taken, if no leave it blanc. | 1. Grains, roots and tubers____________  2. Legumes and nuts_________________  3. Dairy products____________________  4. Flesh food________________________  5. Eggs_____________________________  6. Vitamin A rich fruits and vegetables_________  7. Other fruits and vegetables_________________ | |
| C19. Has your husband ever given you advice about how to feed your baby? | 1. Yes 2. no | |

**Section D; Child morbidity questions**

| D | **Has the child any illness in the past two weeks** | 1. **yes, 0=no** |
| --- | --- | --- |
|  | **If yes, then continue below. Otherwise, skip to the next section**. |  |
| D1. | Cough |  |
| D2. | Difficult or fast breathing |  |
| D3 | Fever |  |
| D4. | Diarrhea |  |
| D5 | Skin rash |  |
| D6. | Ear discharge |  |
| D7 | Eye infection |  |
| D8. | Other (specify ____________) |  |

**Section F: Household Food Insecurity Access Scale and related questions**

|  | **Question** | **Response** |
| --- | --- | --- |
| **F1.** | In the past four weeks, did you worry that your household would not have enough food | ?  1=Yes  0=No |
| **F2.** | If yes, how often did this happen? | 1 = Rarely (once or twice in the past four weeks)  2 = Sometimes (3 to 10 times in the past four weeks)  3 = Often (more than 10 times in the past four weeks) |
| **F3.** | In the past four weeks, were you or any household member not able to eat the kinds of foods you preferred because of a lack of resources? | 1=Yes  0=No |
| **F4.** | If yes, how often did this happen? | 1 = Rarely (once or twice in the past four weeks)  2 = Sometimes (3 to 10 times in the past four weeks)  3 = Often (more than 10 times in the past four weeks) |
| **F5.** | In the past four weeks, did you or any household member have to eat a limited variety of foods due to a lack of resources? | 1=Yes  0=No |
| **F6.** | If yes, how often did this happen? | 1 = Rarely (once or twice in the past four weeks)  2 = Sometimes (3 to 10 times in the past four weeks)  3 = Often (more than 10 times in the past four weeks) |
| **F7.** | In the past four weeks, did you or any household member have to eat some foods that you really did not want to eat because of a lack of resources to obtain other types of food? | 1=Yes  0=No |
| **F8.** | If yes, how often did this happen? | 1 = Rarely (once or twice in the past four weeks)  2 = Sometimes (3 to 10 times in the past four weeks)  3 = Often (more than 10 times in the past four weeks) |
| **F9.** | In the past four weeks, did you or any household member have to eat a smaller meal than you felt you needed because there was not enough food? | 1=Yes  0=No |
| **F10.** | If yes, how often did this happen? | 1 = Rarely (once or twice in the past four weeks)  2 = Sometimes (3 to 10 times in the past four weeks)  3 = Often (more than 10 times in the past four weeks) |
| **F11.** | In the past four weeks, did you or any household member have to eat fewer meals in a day because there was not enough food? | 1=Yes  0=No |
| **F12.** | If yes, how often did this happen? | 1 = Rarely (once or twice in the past four weeks)  2 = Sometimes (3 to 10 times in the past four weeks)  3 = Often (more than 10 times in the past four weeks) |
| **F13.** | In the past four weeks, was there ever no food to eat of any kind in your household because of lack of resources to get food? | 1=yes 0=no |
| **F14.** | If yes, how often did this happen? | 1 = Rarely (once or twice in the past four weeks)  2 = Sometimes (3 to 10 times in the past four weeks)  3 = Often (more than 10 times in the past four weeks) |
| **F15.** | In the past four weeks, did you or any household member go to sleep at night hungry because there was not enough food? | 1=Yes  0=No |
| **F16.** | If yes, how often did this happen? | 1 = Rarely (once or twice in the past four weeks)  2 = Sometimes (3 to 10 times in the past four weeks)  3 = Often (more than 10 times in the past four weeks) |
| **F17.** | In the past four weeks, did you or any household member go a whole day and night without eating anything because there was not enough food? | 1=Yes  0=No |
| **F18.** | If yes, how often did this happen? | 1 = Rarely (once or twice in the past four weeks)  2 = Sometimes (3 to 10 times in the past four weeks)  3 = Often (more than 10 times in the past four weeks) |
| **F 19** | Did you have your own land for farm? | 1. Yes 2. No |
| **F 20** | If yes how much is the size (hectares)  NB: one hectare = Four “Fechasa” | ________ |
| **F 21** | Did you use agricultural input (fertilizer, improved seed, insecticide, pesticide) | 1. Yes 2. No |
| **F 22** | Did you use safety net service | 1. Yes 2. No |
| **F 23** | Did you have access to saving and credit | 1. Yes 2. No |
| **F 25** | Did you use agricultural extension service | 1. Yes 2. No |
| **F 26** | Who is the responsible person to purchase food in your household | 1. Husband 2. spouse |
| **F 27** | How many birr you spend to purchase food from your total income/per month (in kind)? | _____________ |

**Section W. Women Autonomy questions: Ask the mother of the child**

| **W1** | **Freedom of Movement** | 1.Yes | **0.No** |
| --- | --- | --- | --- |
| w1a. | Do you have to ask your husband or a senior family member for permission to go anyplace outside your house or compound? | 1 | 0 |
| W1b | Do you have to ask your husband or a senior family member for permission to go to the local health center? | 1 | 0 |
| W1c | Do you have to ask your husband or a senior family member for permission to go to the local market? | 1 | 0 |
| **W2** | **Decision-Making Regarding Children:**  Please tell me who in your family decides the following: | **1.Wife** | **0.Other** |
| W2a | What to do when a child falls sick? | 1 | 0 |
| W2b | How much schooling to give to your children? | 1 | 0 |
| W2c | To whom to marry your children (probe the mother for current (if exist) and or expectation in future)? | 1 | 0 |
| **W3** | **Household Tasks and Decisions (final say)** | 1 | 0 |
| W3a | What food to buy for family meals | 1 | 0 |
| W3b | Whether to purchase major goods for the household such as oxen, land and house | 1 | 0 |
| **W4** | **Autonomy regarding Family planning service utilization** |  |  |
| W4 | Can you decide the number of children you need to have alone | yes | No |

**Section M. Ask only for the father of the same child the mother responded for. Call the child by their name during the interview**

| **Variable (Male involvement)** | **Response** |
| --- | --- |
| *M1. Availability/Accessibility***:** |  |
| M1. Since CHILD was born, how many months have you lived in the same household as him/her? | *_________________* |
| *M2.Responsibility*: health and finance |  |
| M2a. Have you ever you brought your CHILD to health institution since his birth? | *Yes______________*  *NO______________* |
| M2b. Do you cover financial expenses for your CHILD care? | 1. *Yes________* 2. *NO________* |
| *M3.Engagement*: In feeding and child care |  |
| M3a. In a typical day when you are with your CHILD, do you engage in *diapering, bathing, handling and or recreating your child?* | *Yes________*  *NO________* |
| M3b. How often do you feed your young CHILD? * for children > 6months of age | 1. Always 2. Some times 3. never |

**Section G- Anthropometry**

| **variables** | **Measurements** |
| --- | --- |
| Sex |  |
| Age |  |
| Weight |  |
| Length |  |
| MUAC pregnant mother |  |
| Edema (if any), child |  |
| Edema (if any), mother |  |
| Demi span only for disabled mothers if any |  |

DIETARY DIVE RSITY PROBING TOOL (for question C45)

| IDS QS. NO | list of food groups | Specification for the food group |
| --- | --- | --- |
| 001 | . Cereals | Any bread, rice noodles, biscuits, or any other foods made from millet, sorghum, maize, rice, wheat or teff |
| 002 | Vitamin A rich vegetables and tubers | pumpkin in, carrots, squash, or sweet potatoes that are orange inside + other locally available vitamin-A rich vegetables (e.g. red sweet pepper |
| 003 | White tubers | Any potatoes, yams, manioc, cassava or any other foods made from roots or tubers? |
| 004 | Dark green leafy vegetables | Dark green/leafy vegetables, including wild ones + locally available vitamin-A rich leaves such as amaranth, cassava leaves, kale, spinach etc. |
| 005 | Other vegetables | other vegetables (e.g. tomato, onion, eggplant) ,  including wild vegetables |
| 006 | Vitamin A rich fruits | pumpkin, carrots, squash, or sweet potatoes that are orange inside other locally available vitamin-A rich vegetables (e.g. red sweet pepper) |
| 007 | Other fruits | other fruits, including wild fruits |
| 008 | Organ meat (iron rich) | Liver, kidney, heart or other organ meats or blood-based foods. |
| 009 | Flesh meat | beef, lamb, goat, rabbit, wild game, chicken, duck or other birds |
| 010 | Eggs | chicken, guinea hen or any other egg |
| 011 | Fish | fresh or dried fish or shellfish |
| 012 | Legumes, nuts and seeds | beans, peas, lentils, nuts, seeds or foods made from  these |
| 013 | Milk and milk products | milk, cheese, yogurt or other milk products |
| 014 | Oils and fats (and red palm oil if applicable | oil, fats or butter added to food or used for cooking  Red palm oil, palm nut or palm nut pulp sauce |
